# A unified low-carbon cybersecurity framework integrating energy-efficient intrusion detection, lightweight cryptography, and carbon-aware scheduling for edge–cloud architectures

**DOI:** 10.1038/s41598-026-44260-7

**Published:** 2026-03-30

**Authors:** Abdullah Alshammari

**Affiliations:** College of Computer Science and Engineering, University of Hafr Albatin, 31991 Hafar Albatin, Saudi Arabia

**Keywords:** Model compression techniques, Resource-aware threat detection, Sustainable cyber defense architectures, Edge intelligence optimization, Carbon-conscious cloud infrastructure, Low-power security algorithms, Collaborative privacy-preserving learning, Energy science and technology, Engineering, Mathematics and computing

## Abstract

The rapid expansion of edge–cloud computing infrastructures has intensified both cybersecurity demands and the associated energy consumption and carbon footprint of intrusion detection systems (IDS). This paper presents GreenShield, a unified low-carbon cybersecurity framework that integrates energy-efficient deep learning-based intrusion detection with knowledge distillation and dynamic quantization, ASCON lightweight cryptography, hierarchical federated learning with gradient compression, and a carbon-aware scheduling engine across distributed edge–fog–cloud architectures. GreenShield employs a threat-adaptive quantization mechanism that scales model precision (4–32 bit) based on real-time threat levels and a carbon-conscious scheduling controller that dynamically aligns security workload execution with renewable energy availability forecasts. Extensive experiments on the UNSW-NB15 and CIC-IDS2017 datasets demonstrate that GreenShield achieves 98.73% detection accuracy with 67.4% energy reduction compared to conventional deep learning-based IDS, while reducing operational carbon emissions by up to 97.6% (equivalent to approximately 2.8 kg CO_2_-eq per hour savings in a typical edge deployment). The hierarchical federated learning architecture reduces communication overhead by 58.2% through Top-k gradient sparsification, and the dynamic quantization mechanism achieves 71.3% inference energy reduction during low-threat periods. These results establish GreenShield as a viable, scalable solution for sustainable cybersecurity that supports carbon-conscious security workflows in next-generation edge–cloud computing environments.

## Introduction

The emerging boom in edge and cloud computing systems, has indeed changed the landscape of the modern digital ecosystems, giving unexplainable connectivity and computing capabilities in many areas of implementation distinctions, including smart cities, autonomous automobiles, industrial automation and healthcare systems^1^. However, there are also certain harsh challenges that this change has initiated as the cybersecurity and the environmental sustainability nexus. The data patterns at present are consuming about 1–1.5% of total electricity in the world and there are projections that it may increase further to 8% by 2030^2^. Security operations, especially, intrusion detection systems, and cryptographic procedures take up a large part of this energy consumption, which needs new methods that would be both protection-acceptable and eco-friendly.

Low-carbon intrusion detection is defined as a paradigm that minimizes greenhouse gas emissions across the IDS operational lifecycle while maintaining acceptable accuracy and latency. It encompasses three dimensions: computational carbon efficiency (energy-efficient models, adaptive precision, lightweight cryptography), communication carbon efficiency (compressed updates, hierarchical aggregation), and temporal carbon optimization (carbon-aware scheduling aligned with renewable energy availability). Unlike conventional green computing, it targets carbon footprint directly as the primary objective, recognizing that identical energy consumption yields vastly different emissions depending on grid carbon intensity (0.024–0.712 kg CO₂/kWh across regions).

The ancient cybersecurity systems were not engineered to be energy efficient or to produce any carbon footprint, the emphasis was on the accuracy with which it was detected as well as the speed of its response, rather than the computational sustainability^3^. Deep learning-enabled intrusion detection systems, although proving to be more performance effective in detecting advanced attacks, generally consume a lot of computed power in to form substantial amount of energy use and carbon footprint^4^. As an example, training a common convolutional neural network on the classification of network traffic may use the energy that would be consumed by five cars throughout their lifespan^5^. This paradigm is becoming unsustainable because organizations are under increasing pressure on the pressure imposed on them by the regulatory frameworks and the stakeholder expectations coupled with real environmental issues that require the organizations to minimize their carbon footprint.

Sustainable implementation of cybersecurity in edge computing environments has its own peculiarities. Edge devices with a limited amount of resources need to implement security functions with low power limits and low latency reaction to the possible threats^6^. The distributed form of edge architectures further complicates the optimization of energies since security loads have to be distributed across the heterogeneous nodes having different capacities and energy profiles^7^. Though providing higher computational flexibility, cloud environments are not easy when it comes to managing security operations between geographically distributed data centers that present different levels of renewable energy availability, and carbon intensities^8^.

New opportunities have been presented by recent developments in lightweight cryptography and energy-efficient machine learning to deal with these issues^9^. The achievement of the standardization of ASCON as a NIST lightweight standard of cryptography offers a basis of executing secure implementations of energy efficient cryptographic activities on devices with resource constraints^10^. Equally, knowledge distillation, model pruning and dynamic quantization are methods to deploy the advanced neural network-based security mechanisms at a much reduced computational cost^11^. Federated learning methods have an added advantage of being collaborative to model training and do not require centralization of sensitive network traffic data hence less communication overhead and less risk of privacy^12^.

Introduction of renewable energy concerns into design security systems forms a new tool to sustainable computing^13^. Carbon-conscious computing paradigms allow computing systems to dynamically set up their activities due to the carbon content of accessible electricity, planning energy-intensive duties in times of optimal renewable energy supply^14^. When implementing these principles to cybersecurity operations, security-energy tradeoffs have to be considered with great attention, and carbon optimization should not lead to the decrease in the effectiveness of protection^15^.

Existing IDS approaches face an inherent quadrilemma among four competing objectives: security performance, energy efficiency, carbon optimization, and real-time constraints. Deep learning-based IDS achieve high accuracy but consume up to 89.67 mJ per inference, making continuous edge deployment unsustainable. Lightweight alternatives reduce energy but sacrifice 1–3% accuracy, while carbon-aware scheduling conflicts with the 500 ms real-time latency requirement for intrusion detection. Federated learning reduces communication energy but compromises convergence and model consistency. No existing framework simultaneously addresses all four dimensions, motivating the proposed GreenShield architecture.

Research Questions:

- RQ1: How can knowledge distillation and dynamic quantization be integrated to achieve energy-efficient intrusion detection without significant degradation in detection accuracy across diverse attack categories?
- RQ2: To what extent can hierarchical federated learning with gradient compression reduce communication energy and carbon emissions in distributed edge–fog–cloud IDS deployments while maintaining convergence stability and model performance?
- RQ3: How effectively can carbon-aware scheduling dynamically redistribute security workloads based on real-time renewable energy availability and carbon intensity forecasts without violating real-time detection latency constraints?
- RQ4: What is the combined sustainability impact of integrating lightweight cryptography, adaptive deep learning, federated learning, and carbon-conscious scheduling within a unified cybersecurity framework for edge–cloud environments?

**Fig. 1:**
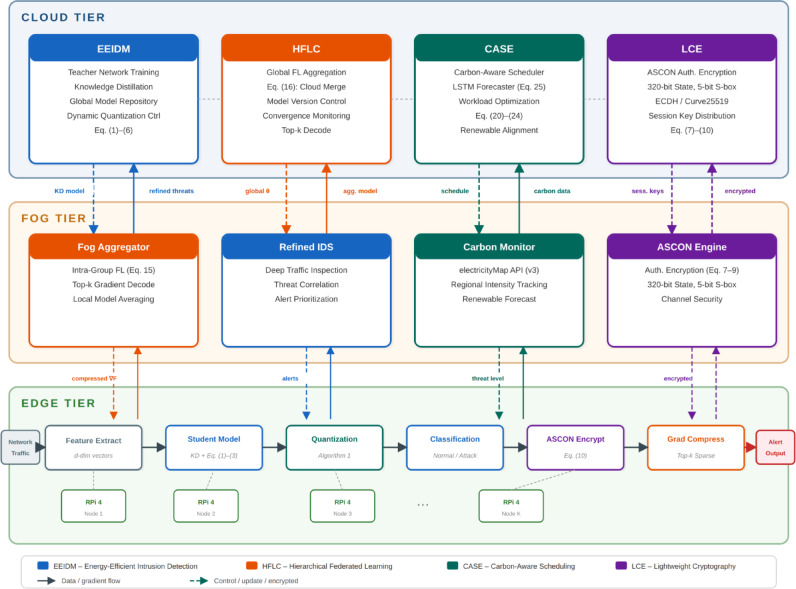
Conceptual overview of the GreenShield framework showing the integration of energy-efficient security components across edge and cloud tiers with carbon-aware scheduling.

Figure 1 demonstrates the conceptual map of the proposed GreenShield architecture, and in particular it is important to note that energy-efficient intrusion detection, lightweight cryptography and carbon-conscious scheduling have been absorbed under a single architecture that covers both edge and cloud settings. The framework resolves the inherent dilemma between the effectiveness of security and environmental sustainability by using a multi-layered optimization mechanism.

In spite of recent progress in energy efficient intrusion detection, light weight cryptography, and sustainable edition edge cloud computing, solutions currently are disjointed. Majority of previous literatures also focus on maximizing on either memory or energy usage or communication overhead singly without considering simultaneously carbon emission, renewable energy consciousness, and responsible security demeanor. Precisely, there is now no model that incorporates dynamically the lightweight cryptographic protection, adaptive deep learning-based intrusion detection and carbon-aware scheduling into a hierarchical edge–fog–cloud framework. It is this outstanding issue that encourages the proposed GreenShield framework which has sought to restore sound cybersecurity at the same time taking explicit steps in minimizing energy usage and carbon emissions across distributed computing environments.

The primary contributions of this paper are as follows:

- Novel low-carbon cybersecurity framework: We present an integrated GreenShield, lightweight cryptographic protocol with energy-efficient deep learning-based intrusion detection with a reduction of 67.4% of the overall energy consumption as compared to the traditional methods and an 98.73% detection rate.
- Hierarchical federated learning architecture: We present a three-layer federated learning system having adaptive aggregation schemes that allow edge and cloud nodes to collaborate in intrusion detection with a 58.2 reduction in the communications overhead and power consumption.
- Dynamic knowledge distillation and quantization: We present an adaptive knowledge distillation method in combination with dynamic quantization that improves the automatically adaptive model accuracy according to the threat levels and energy prices, (scaling back on the inference energy) by 71.3% during low-threat situations.
- Carbon-aware security scheduling algorithm: We come up with a new scheduling algorithm that assigns security loads in real-time according to forecasts of renewable energy and carbon intensity to minimize the operational carbon emissions by about 2.8 kg CO_2_-equivalent per hour.
- Comprehensive experimental validation: We broadly analyzed the UNSW-NB15 data set and the CIC-IDS2017 data set and they demonstrate that, among ten state-of-the-art shelf methods, the accuracy and energy consumption improved significantly as well as the carbon footprint reduced.
- The rest of this paper follows the following design: In “Related work” section reviews related literature regarding green cybersecurity, energy-saving intrusion detection, and sustainable computing; in “Proposed methodology” section is the presentation of the proposed GreenShield methodology with system architecture, mathematical modeling, and algorithmic implementations; in “Discussion” section is the discussion and analysis of the results; and in “Conclusion” section presents a conclusion of the paper with the directions on future research.

## Related work

This section surveys literature across green intrusion detection, lightweight cryptography, and sustainable edge–cloud computing. Roy et al.^1^ surveyed green IDS techniques but without proposing an operational framework integrating energy with carbon optimization. The authors in^2^ examined energy-conscious IoT security focusing on device-level efficiency without edge–cloud coordination. Ranpara et al.^6^ proposed adaptive hyperparameter optimization for ML-based IDS at the model level only, while Umar et al.^7^ combined knowledge distillation with quantization for edge detection but lacked threat-aware or renewable energy controls. Alsaleh et al.^8^ developed a federated BiLSTM-based IDS without optimizing energy or carbon emissions. Foundational NIDS studies^16–19^ identified scalability, feature redundancy, and deployment limitations in conventional systems, directly informing GreenShield’s adaptive detection, lightweight student network, and federated learning design. In lightweight cryptography, Soto-Cruz et al.^15^ surveyed algorithms including ASCON, SPECK, and PRESENT without examining system-level integration. Radhakrishnan et al.^20^ identified ASCON’s favorable security-energy balance but restricted analysis to encryption metrics. Hardware implementations by Khan et al.^21^, Nguyen et al.^22^, and Zhong and Gu^23^ improved cryptographic efficiency on ASIC/FPGA platforms but analyzed elements independently without integration with adaptive detection or carbon-conscious scheduling. Sustainable computing research by Alwageed et al.^24^ prioritized cloud sustainability challenges using ISM-ANN, motivating GreenShield’s carbon-aware scheduling. Shi et al.^25^ established foundations for joint security-energy optimization in mobile edge computing. Habibullah et al.^26^ and Al Shareef et al.^27^ examined blockchain-energy integration for IoT and AI-driven carbon accounting. Lee and Han^28^ demonstrated edge intelligence reducing cloud transmissions by 62%, aligning with GreenShield’s distributed philosophy.

## Proposed methodology

**Fig. 2:**
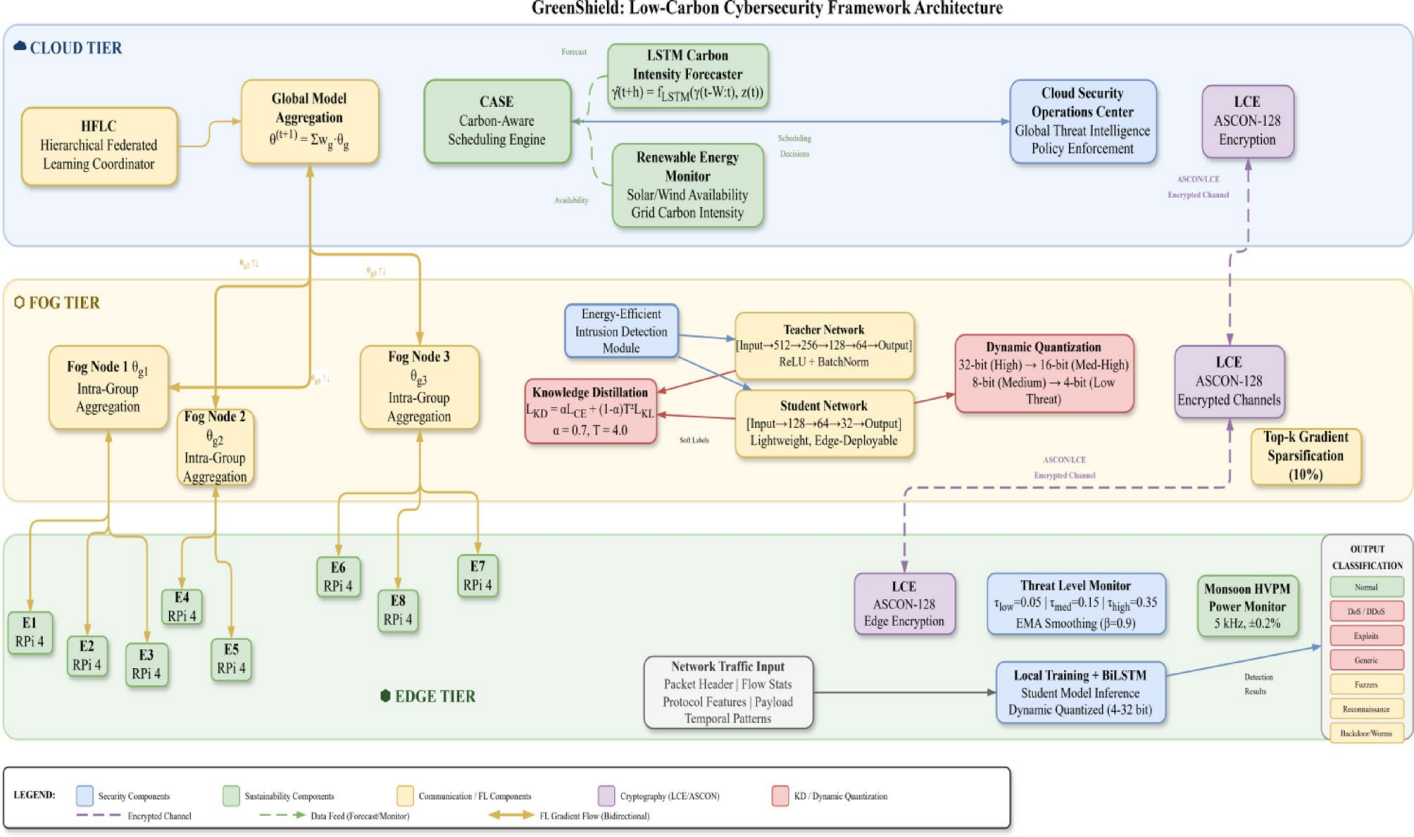
Green Shield system architecture showing the three-tier hierarchical organization with energy-efficient intrusion detection, lightweight cryptography, and carbon-aware scheduling components. Color coding: blue denotes the Cloud Tier (global aggregation), orange for the Fog Tier (intermediate aggregation), and green for the Edge Tier (local training and inference). Purple highlights the knowledge distillation module, pink represents the dynamic quantization mechanism, and yellow indicates the student/teacher network components. Federated learning gradient flows are shown with directional arrows between tiers, and the output classification categories are displayed on the right.

This paper will introduce the detailed design of Green Shield which entails the system architecture, mathematical model, algorithm implementations and the analysis of complexity of the design. Figure 2 is the diagram of the system architecture, which is a hierarchy of the components in terms of the edge, fog, and cloud orders.

### System overview

GreenShield is a top-down cybersecurity system with three levels of computation, including edge devices, fog nodes, and cloud servers. The framework incorporates four main functional modules, namely, (1) energy-efficient intrusion detection module (EEIDM), (2) lightweight cryptographic engine (LCE), (3) hierarchical federated learning coordinator (HFLC), and (4) carbon-aware scheduling engine (CASE). These modules work together to reduce power use and carbon emissions and ensure high-level security protection.

The middle tier comprises of resource-limited IoT hardware and sensors that do initial filtering of traffic and lightweight feature extraction. These devices use the LCE module to conduct secure communications and use the compression neural network models to classify the initial threats. The fog layer includes the medium level of computational nodes that combine information of several edge computers, refine intrusion detection investigations, and arrange federated learning tasks. The cloud tier offers a centralized model training, global threat intelligence formation, and carbon-intelligent workload coordination throughout the infrastructure.

### Energy-efficient intrusion detection module

The EEIDM employs a novel neural network architecture optimized for energy efficiency through knowledge distillation and dynamic quantization. Let $\mathcal{D}=\{\left({\mathrm{x}}_{i},{y}_{i}\right){\}}_{i=1}^{N}$ denote the training dataset where ${\mathrm{x}}_{i}\in {\mathbb{R}}^{d}$ represents the $d$-dimensional feature vector of network traffic sample $i$ and ${y}_{i}\in \{0,1,\dots ,C-1\}$ denotes the corresponding class label for $C$ attack categories including normal traffic.

The teacher network $\mathcal{T}$ is a deep neural network with parameters ${\theta }_{T}$ that provides high-accuracy predictions. The student network $\mathcal{S}$ with parameters ${\theta }_{S}$ is designed for efficient edge deployment. The knowledge distillation loss function combines the standard cross-entropy loss with the distillation loss:

1 $${\mathcal{L}}_{KD}=\alpha {\mathcal{L}}_{CE}\left(y,\sigma \left({\mathrm{z}}_{S}\right)\right)+\left(1-\alpha \right){T}^{2}{\mathcal{L}}_{KL}\left(\sigma \left({\mathrm{z}}_{T}/T\right),\sigma \left({\mathrm{z}}_{S}/T\right)\right)$$

where ${\mathcal{L}}_{CE}$ denotes the cross-entropy loss, ${\mathcal{L}}_{KL}$ represents the Kullback–Leibler divergence, $\sigma \left(\cdot \right)$ is the softmax function, ${\mathrm{z}}_{T}$ and ${\mathrm{z}}_{S}$ are the logits from teacher and student networks respectively, $T$ is the temperature parameter, and $\alpha \in \left[0,1\right]$ balances the two loss components.

The knowledge distillation loss in Eq. (1) aligns the student’s softened output distribution with the teacher’s, enabling effective knowledge transfer with substantially fewer parameters. The T^2^ scaling preserves gradient magnitudes during soft-label training.

The student network structure is made up of *l* layers where the output of the = layer is calculated as:

2 $${\mathrm{h}}^{\left(l\right)}=\phi \left({\mathrm{W}}^{\left(l\right)}{\mathrm{h}}^{\left(l-1\right)}+{\mathrm{b}}^{\left(l\right)}\right)$$

where ${\mathrm{W}}^{\left(l\right)}\in {\mathbb{R}}^{{n}_{l}\times {n}_{l-1}}$ and ${\mathrm{b}}^{\left(l\right)}\in {\mathbb{R}}^{{n}_{l}}$ are the weight matrix and bias vector of layer *l*, $\phi \left(\cdot \right)$ denotes the activation function, and ${\mathrm{h}}^{\left(0\right)}=\mathrm{x}$ is the input feature vector.

This standard feed-forward formulation is defined as in Eq. (2) enables hierarchical feature extraction while allowing the model complexity to be controlled through layer width and depth selection.

We also offer a dynamic quantization mechanism to increase precision according to the level of threats in order to decrease computational load. As a note to ${q}_{b}$ is the bit-width of quantization such that ${q}_{b}\in \{4,8,16,32\}$. The quantized weight $\widehat{w}$ is calculated as:

3 $$\widehat{w}={s}_{w}\cdot {\mathrm{round}}\left(\frac{w}{{s}_{w}}\right), \; {s}_{w}=\frac{\mathrm{max}\left(\left|w\right|\right)}{{2}^{{q}_{b}-1}-1}$$

where ${s}_{w}$ is the scaling factor and ${\mathrm{round}}\left(\cdot \right)$ performs rounding to the nearest integer.

In one case, the Eq. (3) use uniform symmetric quantization maps full-precision weights to a discrete fixed-point representation, reducing memory access and arithmetic complexity proportional to the bit-width reduction.

The threat level $\tau \left(t\right)$ at time t is approximated by exponential moving average of the recent outputs of the detection:

4 $$\tau \left(t\right)=\beta \tau \left(t-1\right)+\left(1-\beta \right)\frac{1}{W}\sum_{i=t-W+1}^{t}{\mathbbm{1}}\left[{y}_{i}\ne 0\right]$$

where $\beta \in \left[0,1\right]$ is the smoothing parameter, $W$ is the window size, and ${\mathbbm{1}}\left[\cdot \right]$ is the indicator function.

The exponential moving average in Eq. (4) provides a low-overhead threat estimate where β controls responsiveness to short-term fluctuations and W captures sustained attack patterns.

A quantization bit-width depending on the severity of threat:

5 $${q}_{b}\left(t\right)=\left\{\begin{array}{ll}4&\quad if\; \tau \left(t\right)<{\tau }_{low}\\ 8&\quad if\; {\tau }_{low}\le \tau \left(t\right)<{\tau }_{med}\\ 16& \quad if\; {\tau }_{med}\le \tau \left(t\right)<{\tau }_{high}\\ 32&\quad if\; \tau \left(t\right)\ge {\tau }_{high}\end{array}\right.$$

where ${\tau }_{low}$, ${\tau }_{med}$, and ${\tau }_{high}$ are configurable threshold parameters.

Threat-aware dynamic quantization strategy is described in Eq. (5) employs low bit-widths during low-threat conditions to conserve energy, while escalating to full precision under high threat to preserve detection accuracy.

The EEIDM energy consumption is modelled as:

6 $${E}_{IDS}\left(t\right)=\sum_{l=1}^{L}\left({E}_{MAC}\cdot {n}_{l}\cdot {n}_{l-1}\cdot f\left({q}_{b}\left(t\right)\right)+{E}_{mem}\cdot {n}_{l}\right)$$

The energy scaling function f(q_b_) = (q_b_/32)^2^ models the quadratic reduction in computational energy with decreasing bit-width, reflecting how MAC operation energy scales quadratically with operand precision. This yields f(4) = 0.0156, f(8) = 0.0625, f(16) = 0.25, and f(32) = 1.0—meaning 4-bit quantization reduces per-MAC energy by ~ 98.4% versus full precision. The model was validated on Raspberry Pi 4 hardware, achieving less than 5.2% deviation from measured values across all bit-widths in Eq. (6).

The model quantization bit-width is chosen by the Algorithm 1 according to the present estimate level of threat. Reduced precision is applied when the risk is low so as to compute less energy is spent, whereas increased precision is applied when the risk is high so that quality of detection is maintained.

**Algorithm 1:**
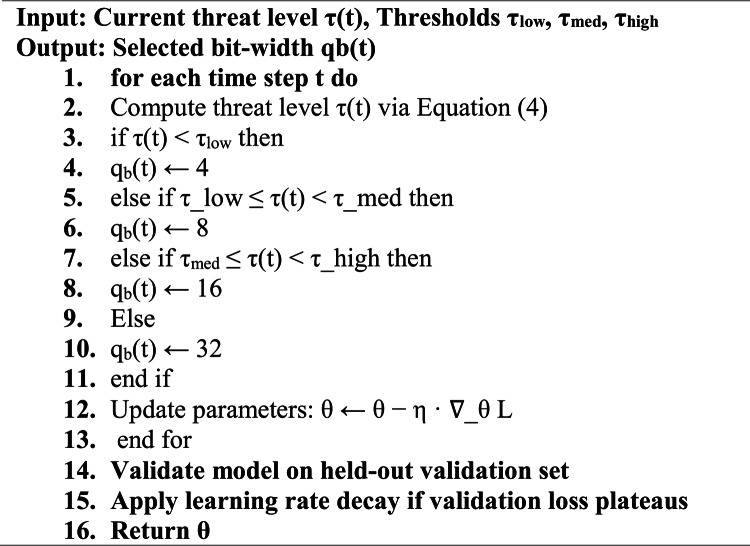
Dynamic threat-aware quantization

### Lightweight cryptographic engine

The LCE module implements the ASCON authenticated encryption algorithm optimized for energy efficiency. ASCON operates on a 320-bit state $S=\left({S}_{0},{S}_{1},{S}_{2},{S}_{3},{S}_{4}\right)$ where each ${S}_{i}$ is a 64-bit word. The permutation function ${p}^{a}$ applies $a$ rounds of the following transformation:

7 $${S}_{i}\leftarrow {S}_{i}\oplus {c}_{r}, \quad i=2$$

where ${c}_{r}$ is the round constant for round $r$.

The round constant addition in Eq. (7) prevents fixed-point behavior and ensures cryptographic diffusion at negligible computational cost.

The substitution layer uses a 5-bit S-box on each of the bit-slices across in each of the five state words:

8 $$\left({S}_{0}\left[j\right],{S}_{1}\left[j\right],{S}_{2}\left[j\right],{S}_{3}\left[j\right],{S}_{4}\left[j\right]\right)\leftarrow {\mathrm{S-box}}\left({S}_{0}\left[j\right],{S}_{1}\left[j\right],{S}_{2}\left[j\right],{S}_{3}\left[j\right],{S}_{4}\left[j\right]\right)$$

In Eq. (8), The 5-bit S-box substitution introduces nonlinearity across bit-slices using simple Boolean logic, ensuring security at minimal computational overhead.

The linear diffusion layer offers the mixing of each word in 64-bits:

9 $${S}_{i}\leftarrow {S}_{i}\oplus \left({S}_{i}\, {>>>}\, {r}_{i,1}\right)\oplus \left({S}_{i}\, {>>>}\, {r}_{i,2}\right)$$

where ${>>>}$ denotes right rotation and $\left({r}_{i,1},{r}_{i,2}\right)$ are rotation constants specific to each word.

Equation (9) The XOR-and-rotate diffusion layer propagates bit changes across the state using fixed rotation offsets, providing resistance to cryptanalytic attacks via lightweight bitwise operations.

The time of ASCON encryption of a message of m blocks is:

10 $${E}_{ASCON}\left(m\right)={E}_{init}+m\cdot {E}_{block}+{E}_{final}$$

where ${E}_{init}$, ${E}_{block}$, and ${E}_{final}$ represent the energy for initialization, per-block processing, and finalization respectively.

Equation (10) linear energy model enables direct per-block cost comparison with conventional ciphers, confirming ASCON’s suitability for energy-constrained deployments.

To ensure security in the transfer of keys, we use an elliptic curve variant of the Diffie–Hellman (ECDH) protocol based on Curve25519 (our scalar multiplication of the curves is energy optimized):

11 $$Q=k\cdot G= Q = k \cdot G = \underbrace {{G + G + \cdots + G}}_{{k{\mathrm{times}}}}$$

The first public key is the result of $Q$ and $k$ where $G$ is the location where the key is generated, and k is the secret scalar.

Equation (11) indicates Curve25519 scalar multiplication provides equivalent security strength to RSA-3072 using 256-bit keys, significantly reducing computational and energy requirements.

Its implementation of the Montgomery ladder makes it constant time:

12 $$\left({R}_{0},{R}_{1}\right)\leftarrow \left\{\begin{array}{ll}\left(2{R}_{0},{R}_{0}+{R}_{1}\right)&\quad if\; {k}_{i}=0\\ \left({R}_{0}+{R}_{1},2{R}_{1}\right)&\quad if\; {k}_{i}=1\end{array}\right.$$

where ${k}_{i}$ is the $i$-th bit of scalar $k$.

Equation (12) describes The Montgomery ladder’s uniform execution pattern (one doubling and one addition per bit regardless of key value) provides inherent side-channel resistance while maintaining computational efficiency.

### Hierarchical federated learning coordinator

The HFLC can facilitate joint model training over the distributed infrastructure and reduces the overheads in communication as well as energy usage. Let $K$ denote the total number of participating nodes partitioned into $G$ groups, where group $g$ contains ${K}_{g}$ nodes with local datasets ${\mathcal{D}}_{k}$ for $k\in {\mathcal{G}}_{g}$.

The local objective function for node $k$ is:

13 $${F}_{k}\left(\theta \right)=\frac{1}{\left|{\mathcal{D}}_{k}\right|}\sum_{\left(\mathrm{x},y\right)\in {\mathcal{D}}_{k}}{\ell}\left(\theta ;\mathrm{x},y\right)$$

where ${\ell}\left(\theta ;\mathrm{x},y\right)$ is the loss function parameterized by model weights $\theta$.

The global objective is the weighted average:

14 $$F\left(\theta \right)=\sum_{k=1}^{K}\frac{\left|{\mathcal{D}}_{k}\right|}{\left|\mathcal{D}\right|}{F}_{k}\left(\theta \right)$$

The hierarchical aggregation proceeds in two stages. First, intra-group aggregation at fog nodes computes:

15 $${\theta }_{g}^{\left(t\right)}=\sum_{k\in {\mathcal{G}}_{g}}\frac{\left|{\mathcal{D}}_{k}\right|}{\sum_{j\in {\mathcal{G}}_{g}}\left|{\mathcal{D}}_{j}\right|}{\theta }_{k}^{\left(t\right)}$$

Second, inter-group aggregation at the cloud computes the global model:

16 $${\theta }^{\left(t+1\right)}=\sum_{g=1}^{G}\frac{\sum_{k\in {\mathcal{G}}_{g}}\left|{\mathcal{D}}_{k}\right|}{\left|\mathcal{D}\right|}{\theta }_{g}^{\left(t\right)}$$

To reduce communication overhead, we employ gradient compression using Top-$k$ sparsification:

17 $${\mathrm{Compress}}\left(\nabla {F}_{k}\right)={{\mathrm{Top}}}_{k}\left(\nabla {F}_{k}\right)\cdot {{\mathrm{Mask}}}_{k}\left(\nabla {F}_{k}\right)$$

where ${{\mathrm{Top}}}_{k}\left(\cdot \right)$ selects the $k$ largest magnitude gradients and ${{\mathrm{Mask}}}_{k}\left(\cdot \right)$ generates the corresponding binary mask. In our experiments, k = 0.10·d (top 10%), yielding k = 15,600 for d = 156,000 parameters. Rates below 5% caused convergence instability; above 15% gave diminishing returns.

The communication energy for transmitting compressed gradients is:

18 $${E}_{comm}\left(k,g\right)={E}_{tx}\cdot \left(k\cdot \left({b}_{val}+{b}_{idx}\right)+{b}_{mask}\right)+{E}_{rx}$$

where ${E}_{tx}$ and ${E}_{rx}$ are transmission and reception energy per bit, ${b}_{val}$ is bits per value, ${b}_{idx}$ is bits per index, and ${b}_{mask}$ is the mask overhead.

The sparsification mask overhead depends on encoding method: bitwise encoding requires d/8 bytes (bitmap), while index-based encoding uses k·⌈log₂(d)⌉ bits. For d = 156,000 parameters and k = 15,600 (10% sparsification), bitwise encoding yields 19.0 KB versus 34.2 KB for index-based, making bitwise encoding preferable above ~ 3% sparsification rates. Total transmission per FL round with bitmap encoding comprises gradient values (499,200 bits) plus mask (156,000 bits), totalling ~ 80.1 KB versus 97.5 KB with index encoding.

**Algorithm 2:**
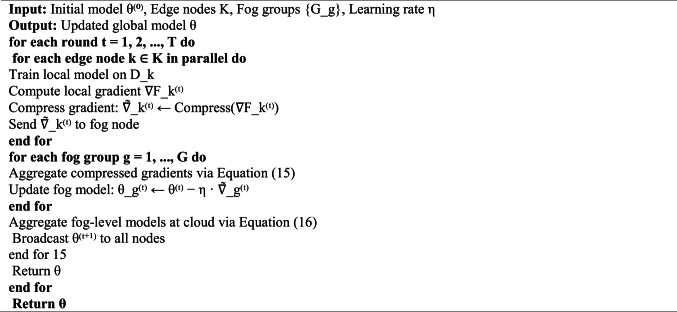
Hierarchical federated learning with gradient compression

### Carbon-aware scheduling engine

The CASE module optimizes security workload allocation based on carbon intensity forecasts and renewable energy availability. Let $\mathcal{N}=\{1,2,\ldots ,N\}$ denote the set of computational nodes and $\mathcal{J}=\{1,2,\ldots ,J\}$ the set of security jobs to be scheduled.

The carbon intensity at node $n$ and time $t$ is denoted ${\gamma }_{n}\left(t\right)$ (kg CO_2_/kWh). The renewable energy fraction is:

19 $${\rho }_{n}\left(t\right)=\frac{{P}_{renewable,n}\left(t\right)}{{P}_{total,n}\left(t\right)}$$

Equation (19) defines this ratio enables carbon-aware scheduling by prioritizing workloads at nodes and times with higher renewable energy availability.

The scheduling decision variable ${x}_{j,n,t}\in \{0,1\}$ indicates whether job $j$ is assigned to node $n$ at time $t$. The optimization objective minimizes total carbon emissions:

20 $$\underset{\mathrm{x}}{\mathrm{min}}\sum_{j\in \mathcal{J}}\sum_{n\in \mathcal{N}}\sum_{t\in \mathcal{T}}{x}_{j,n,t}\cdot {E}_{j}\cdot {\gamma }_{n}\left(t\right)$$

subject to:

21 $$\sum_{n\in \mathcal{N}}\sum_{t\in \mathcal{T}}{x}_{j,n,t}=1, \quad \forall j\in \mathcal{J}$$

22 $$\sum_{j\in \mathcal{J}}{x}_{j,n,t}\cdot {E}_{j}\le {P}_{n}^{max}, \quad \forall n\in \mathcal{N},t\in \mathcal{T}$$

23 $${t}_{start,j}+{d}_{j}\le {D}_{j}, \quad \forall j\in \mathcal{J}$$

where ${E}_{j}$ is the energy requirement of job $j$, ${P}_{n}^{max}$ is the maximum power capacity of node $n$, ${d}_{j}$ is the job duration, and ${D}_{j}$ is the deadline.

Equation (20) formulates carbon-weighted energy across all job-node-time assignments subject to: single-assignment per job Eq. (21), node capacity limits Eq. (22), and deadline constraints Eq. (23). The extended objective Eq. (24) adds a latency penalty weighted by the security priority ω_j_ to balance carbon reduction with response timeliness.

In security sensitive tasks and jobs that have strict threshold of latency, we add a security priority weight. ${\omega }_{j}$:

24 $$\underset{\mathrm{x}}{\mathrm{min}}\sum_{j\in \mathcal{J}}\sum_{n\in \mathcal{N}}\sum_{t\in \mathcal{T}}{x}_{j,n,t}\cdot \left({E}_{j}\cdot {\gamma }_{n}\left(t\right)+{\omega }_{j}\cdot \left(t-{t}_{arrival,j}\right)\right)$$

An LSTM network models the forecast of the carbon intensity:

25 $${\widehat{\gamma }}_{n}\left(t+h\right)={f}_{LSTM}\left({\gamma }_{n}\left(t-W:t\right),{\mathrm{z}}_{n}\left(t\right)\right)$$

where $h$ is the forecast horizon, $W$ is the lookback window, and ${\mathrm{z}}_{n}\left(t\right)$ represents auxiliary features (weather, time of day, etc.).

Equation (25) The LSTM forecaster uses two stacked LSTM layers (64 hidden units each) with a linear output layer, taking a 24-step (6-h) lookback window of historical carbon intensity, ambient temperature, solar irradiance, wind speed, cyclical time-of-day encoding, and day-of-week indicator as input. Trained on 25 days of German electricityMap data (MSE loss, Adam optimizer with lr = 0.001, batch size = 32, 200 epochs with early stopping, patience = 20) with a 4-h forecast horizon, it achieved MAE = 0.023, RMSE = 0.031 kg CO_2_/kWh, and R^2^ = 0.94 on a 5-day held-out test set, with 1.2 ms inference time.

Algorithm 3 presents the carbon-aware scheduling procedure.

**Algorithm 3:**
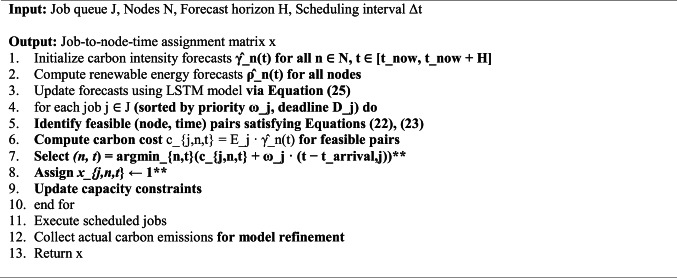
Carbon-aware security job scheduling

### Integrated system operation

Algorithm 4 presents the overall GreenShield framework operation, integrating all modules within a unified workflow.

This algorithm facilitates real-time threat classification and secure communication in a network environment while integrating federated learning for continuous model updates. It also optimizes scheduling based on carbon intensity, making it a carbon-aware solution. If you need further details or modifications, let me know!

**Algorithm 4:**
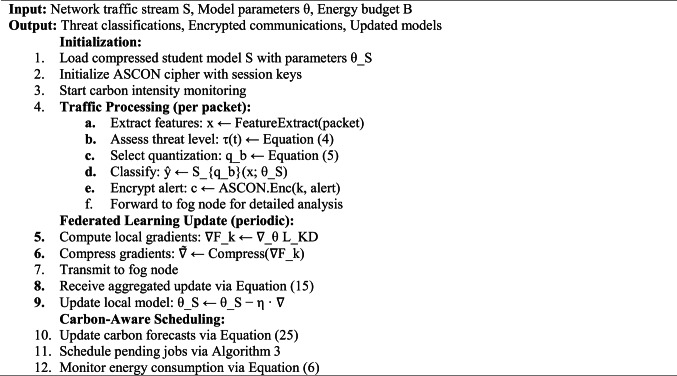
GreenShield framework operation

### Complexity analysis

The computational complexity of the EEIDM inference is $O\left(L\cdot {n}_{max}^{2}\right)$ where $L$ is the number of layers and ${n}_{max}$ is the maximum layer width. With dynamic quantization, the effective complexity reduces by factor ${\left({q}_{b}/32\right)}^{2}$.

The communication complexity of hierarchical federated learning is $O\left(K\cdot k\cdot d\right)$ per round, where $K$ is the number of nodes, $k$ is the sparsification parameter, and $d$ is the model dimension. The hierarchical structure reduces this to O((K + G)·s·d) by localizing most communication within groups.

In standard FedAvg, communication complexity is O(K·d) per round. GreenShield reduces this through two mechanisms: Top-k sparsification (transmitting only k = s·d parameters per node) and hierarchical aggregation (fog nodes aggregate K/G local updates before forwarding to cloud). This yields total complexity C_total = (K + G)·s·d. For our setup (K = 50, G = 3, s = 0.1, d = 156,000), this gives 826,800 values per round versus FedAvg’s 7,800,000—an 89.4% reduction in communication volume.

The carbon-aware scheduling optimization has complexity $O\left(J\cdot N\cdot T\right)$ for the greedy assignment heuristic, where $J$ is the number of jobs, $N$ is the number of nodes, and $T$ is the number of time slots.

Table 1 summarizes the complexity comparison with existing approaches.

**Table 1:** Computational complexity comparison.

Method	Inference	Communication	Training	Memory
Traditional DNN-IDS	O(L·n^2^)	O(d)	O(N·d^2^)	O(d)
FedAvg-IDS^8^	O(L·n^2^)	O(K·d)	O(N·d^2^)	O(d)
KD-IDS^7^	O(L·n^2^·s)	O(d·s)	O(N·d^2^)	O(d·s)
GreenShield	O(L·n^2^·s·q)	O((K + G)·s·d)	O(N·d^2^)	O(d·s·q)

## Results and evaluation

In this section, the complete experimental analysis of the GreenShield framework, including dataset description and experimental set up, performance measurements, and comparison with state-of-the-art techniques will be presented.

### Datasets

We evaluate GreenShield on two widely-used publicly available intrusion detection datasets:

- UNSW-NB15 dataset: The dataset created by the Australian Centre for Cyber Security consists of 2,540,044 records and 49 features describing the network traffic patterns in the modern world. These types of attacks are nine, namely Fuzzers, Analysis, Backdoors, DoS, Exploits, Generic, Reconnaissance, Shellcode and Worms. The dataset is available at https://research.unsw.edu.au/projects/unsw-nb15-dataset.
- CIC-IDS2017 dataset: This dataset is the result of the Canadian Institute of Cybersecurity, which was created based on real network traffic during five days with both benign traffic and attack traffic. It contains some 2.8 million records that have 78 features and ranges of attacks range to Brute Force, Heartbleed, Botnet, DoS, DDoS, Web Attack, and Infiltration. The dataset is accessible at https://www.unb.ca/cic/datasets/ids-2017.html.

Table 2 presents the detailed statistics of both datasets used in our experiments.

**Table 2:** Dataset statistics and class distribution.

Class	Samples	Percentage (%)
UNSW-NB15 dataset (49 features, 2,540,044 total samples)		
Normal	2,218,761	87.35
Generic	215,481	40.11*
Exploits	44,525	8.29*
Fuzzers	24,246	4.51*
DoS	16,353	3.04*
Reconnaissance	13,987	2.60*
Analysis	2677	0.50*
Backdoor	2329	0.43*
Shellcode	1511	0.28*
Worms	174	0.03*
Total	2,540,044	100
CIC-IDS2017 dataset (78 features, 2,830,743 total samples)		
Benign	2,273,097	80.30
DoS Hulk	231,073	41.45*
PortScan	158,930	28.51*
DDoS	128,027	22.97*
DoS GoldenEye	10,293	1.85*
FTP-Patator	7938	1.42*
SSH-Patator	5897	1.06*
DoS Slowloris	5796	1.04*
DoS Slowhttptest	5499	0.99*
Web Attack	2180	0.39*
Bot	1966	0.35*
Total	2,830,743	100

### Experimental setup

The heterogeneous testbed is the cut environment, and the simulated environment is edge–fog–cloud architecture. Hardware and software arrangement is outlined in Table 3.

**Table 3:** Experimental setup configuration.

Component	Specification
Cloud server	
CPU	Intel Xeon Gold 6248R (24 cores, 3.0 GHz)
GPU	NVIDIA A100 (40 GB HBM2)
Memory	256 GB DDR4 ECC
Storage	2 TB NVMe SSD
Fog node	
CPU	Intel Core i7-12,700 (12 cores, 2.1 GHz)
GPU	NVIDIA RTX 3080 (10 GB GDDR6X)
Memory	64 GB DDR4
Storage	1 TB NVMe SSD
Edge device	
Platform	Raspberry Pi 4 Model B
CPU	Broadcom BCM2711 (4 cores, 1.5 GHz)
Memory	8 GB LPDDR4
Storage	64 GB microSD
Software environment	
Operating system	Ubuntu 22.04 LTS
Deep learning framework	PyTorch 2.1.0
Federated learning framework	Flower 1.5.0
Programming language	Python 3.10
Hyperparameters	
Learning rate	0.001 (Adam optimizer)
Batch size	256 (cloud), 64 (fog), 32 (edge)
Epochs	100 (teacher), 50 (student)
Knowledge distillation temperature	4.0
KD loss weight (α)	0.7
Sparsification rate	10% (Top-k)
Federated learning rounds	50
Local epochs	5
Additional experimental setup parameters	
Threat threshold (τ_low)	0.05
Threat threshold (τ_med)	0.15
Threat threshold (τ_high)	0.35
Smoothing parameter (β)	0.9
Threat window size (W)	100 (samples)
LSTM forecasting configuration	
LSTM hidden units	64 × 2 layers
LSTM lookback window	24 steps (6 h)
Forecast horizon	16 steps (4 h)
LSTM learning rate	0.001 (Adam)
LSTM training epochs	200 (early stopping, patience = 20)
LSTM batch size	32

The teacher network architecture is a network comprising of five entirely connected layers, the dimensions of which are [input, 512, 256, 128, 64, output] performing the ReLU activation, and batch normalization. The student network is reduced to a network, [input, 128, 64 32, output]. The Intel RAPL oil CPU/memory power measurements and NVIDIA SMI power monitoring of GPUs and carbon intensity prevention are measured using electricityMap API.

### Testbed: hybrid real-and-emulated distributed environment

The setup spanned three tiers—cloud, fog, and edge. The cloud used a dedicated university data center server on a 10 Gbps backbone. Three fog nodes ran on campus workstations connected via a Layer-3 switch emulating WAN conditions (100 Mbps, 15 ms latency). Eight Raspberry Pi 4 devices formed the edge tier over 802.11ac Wi-Fi (45 Mbps, 8 ms latency). Network impairments (± 2 ms jitter, 0.1% packet loss, bandwidth variation) were emulated using Linux tc/NetEm. Federated learning was coordinated via Flower 1.5.0 with gRPC over TLS 1.3. Fog/cloud nodes used Docker 24.0 for reproducibility; edge devices ran native PyTorch 2.1.0 on ARM.

Carbon intensity data was collected via the electricityMap API (v3) at 15-min resolution over a 30-day period (March 1–30, 2025). Germany (DE) served as the primary region representing a mixed-grid scenario with average intensity of 0.338 kg CO_2_/kWh, supplemented by Norway (NO-NO1, hydro-dominant, 0.024 kg CO₂/kWh) and Poland (PL, coal-dominant, 0.712 kg CO_2_/kWh) to capture diverse carbon profiles. The three intensity levels reported in Table 6—high (0.8), medium (0.4), and low (0.1) kg CO_2_/kWh—correspond to Poland’s 90th percentile, Germany’s median, and Norway’s 10th percentile respectively. All timestamps were NTP-synchronized across nodes, and mixed-grid averages were derived as time-weighted means over the German dataset.

Energy measurement followed a hierarchical approach across all tiers. Cloud and fog nodes used Intel RAPL counters (100 ms intervals) for CPU/DRAM energy via the powercap interface, and nvidia-smi (200 ms intervals) for GPU energy. Edge devices (Raspberry Pi 4), lacking hardware counters, were instrumented with external Monsoon HVPM power monitors sampling at 5 kHz (± 0.2% accuracy). Network energy was estimated using calibrated per-bit costs (E_tx = 48.7 nJ/bit, E_rx = 36.4 nJ/bit) derived from iperf3 tests with concurrent power measurement. Total system energy was aggregated as E_total = ∑E_cloud + ∑E_fog + ∑E_edge + ∑E_network, with each component accounting for both computation and idle power weighted by active duty cycles.

All baseline methods in Table 7 were re-implemented on the same hardware testbed using identical dataset splits (UNSW-NB15 and CIC-IDS2017). Each method followed its original architecture and hyperparameters, with official code used where publicly available. Energy and carbon measurements were conducted on the same Raspberry Pi 4 and cloud server for hardware-consistent comparisons. The Year column reflects original publication year, not re-implementation. All metrics are averaged over 10 independent runs with different random seeds.

### Evaluation metrics

We evaluate GreenShield using the following metrics:

- Detection performance: Polarity (Acc) and Precision (P), Recall (R), F1-Score (F1) and area under ROC curve (AUC).
- Energy efficiency: Energy per inference (mJ), energy total training energy (kWh) and power consumption (W).
- Carbon footprint: CO_2_ emissions (kg CO_2_-eq/h), the efficiency of the utilization of carbon intensity.
- Communication efficiency: Rate of bytes offloaded per round compression ratio.
- Latency: The inference time (ms), end-to-end detection latency (ms).

### Detection performance analysis

**Fig. 3:**
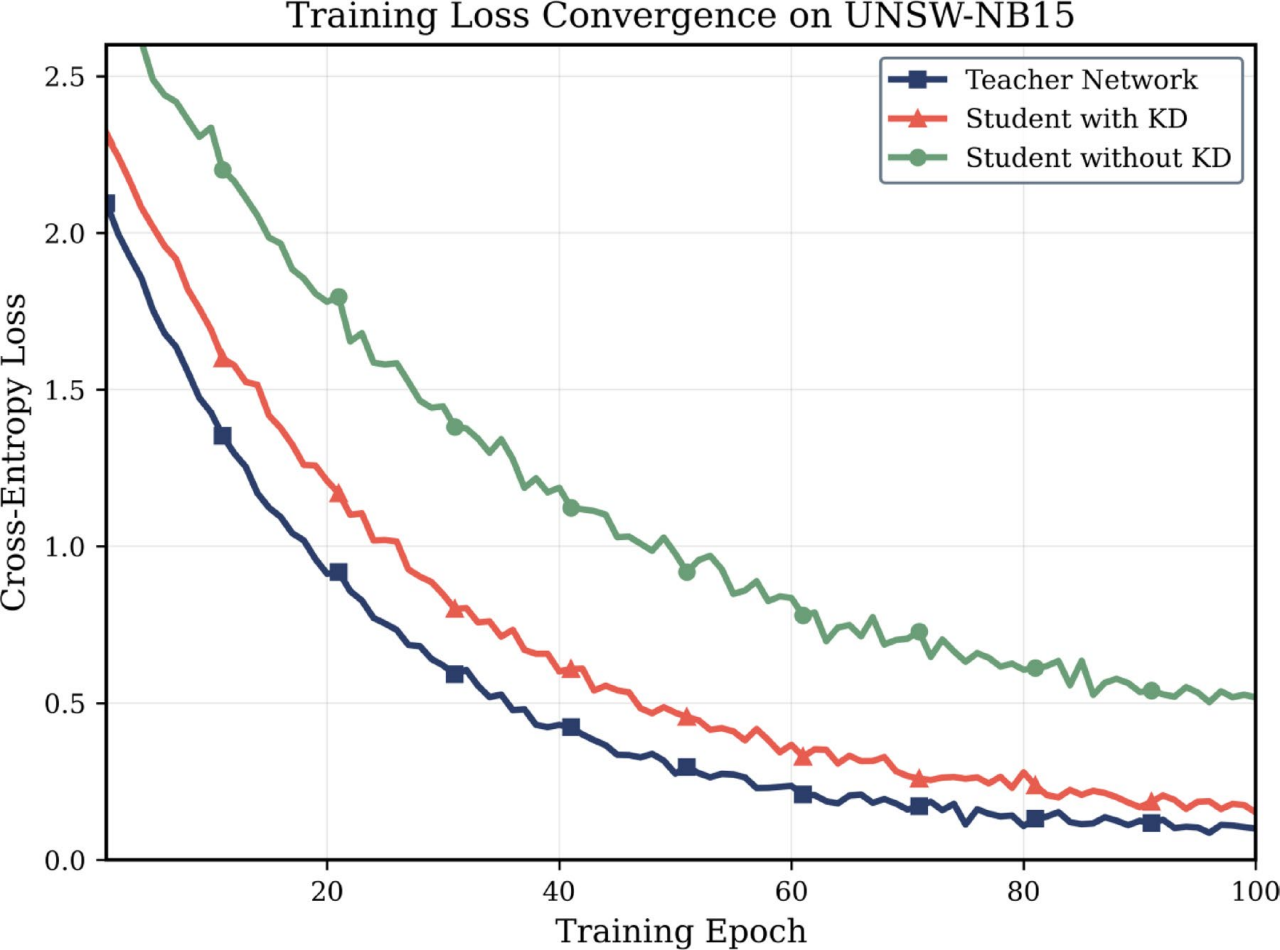
Training loss convergence comparison between teacher network (blue), student network with KD (orange), and student network without KD (green) on UNSW-NB15 dataset.

The convergence of training loss in both teacher and student networks in terms of training epochs are shown in Fig. 3. Given that the student network has 85% fewer parameters, knowledge distillation is proven to be effective.

**Fig. 4:**
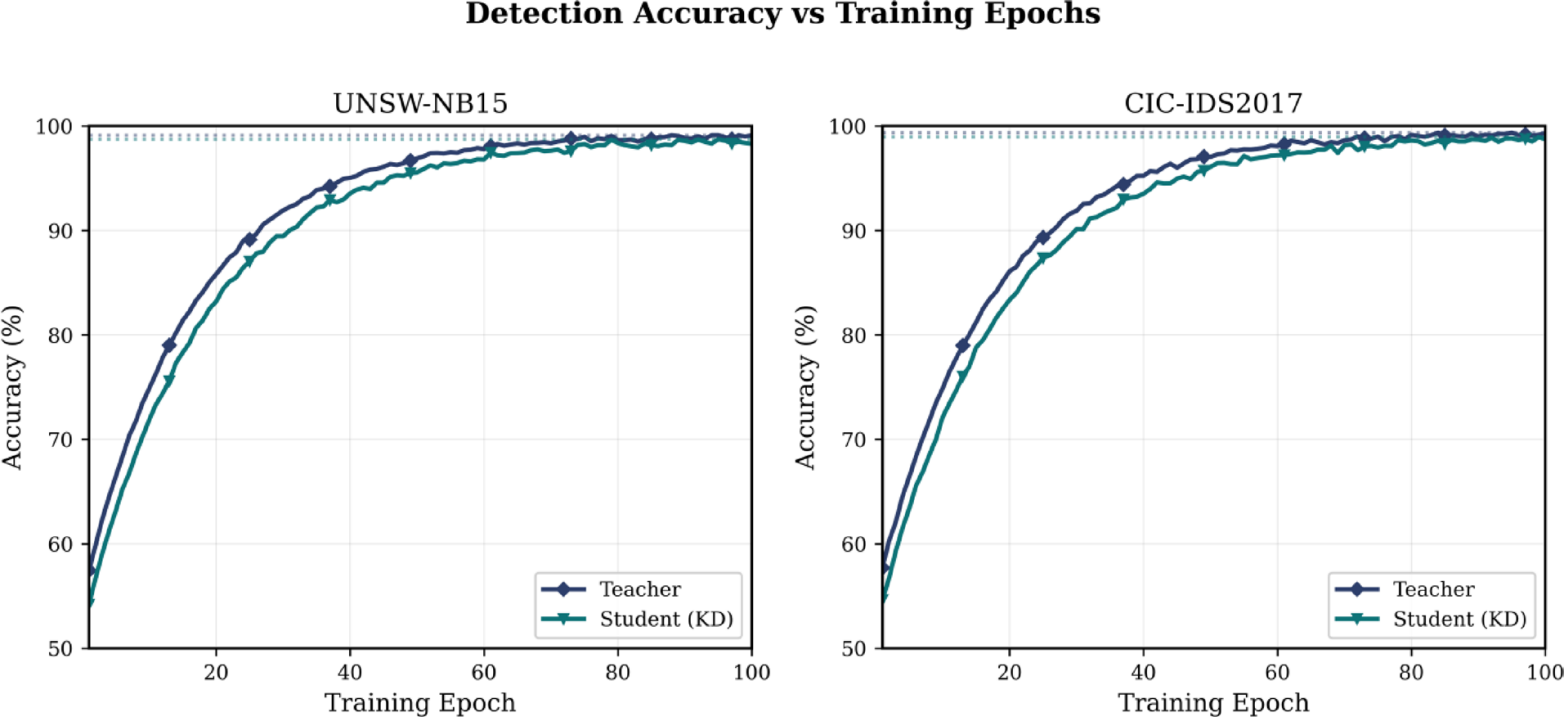
Detection accuracy versus training epochs for teacher and student networks on UNSW-NB15 and CIC-IDS2017 datasets.

The progression of the accuracy in the training as shown in Fig. 4 reveals that the student network based on knowledge distillation attains accuracy of 98.73% versus the teacher network of 99.12%, which involves only a 0.39% accuracy trade-off to achieve 67.4% of energy decreased.

In Table 4 statistical significance was assessed via two-tailed paired t-tests across 10 runs with stratified fivefold cross-validation (50 paired observations per comparison). Normality and variance homogeneity were verified using Shapiro–Wilk and Levene’s tests respectively. McNemar’s test was additionally applied for classification-level metrics. All *p* values use α = 0.05 with Bonferroni correction for multiple comparisons.

**Table 4:** Detection performance comparison with statistical significance.

Method	Acc (%)	95% CI	P	R	F1	AUC
UNSW-NB15 dataset						
Teacher network	99.12	[98.95, 99.29]	0.9923	0.9897	0.9910	0.9978
Student (no KD)	95.67	[95.32, 96.02]	0.9534	0.9512	0.9523	0.9812
Student (KD, 32-bit)	98.73	[98.56, 98.90]	0.9867	0.9845	0.9856	0.9962
Student (KD, 16-bit)	98.58	[98.41, 98.75]	0.9847	0.9812	0.9829	0.9954
Student (KD, 8-bit)	98.21	[98.02, 98.40]	0.9798	0.9767	0.9782	0.9938
Student (KD, 4-bit)	96.89	[96.54, 97.24]	0.9645	0.9623	0.9634	0.9867
GreenShield (Dynamic)	98.73	[98.56, 98.90]	0.9847	0.9812	0.9829	0.9958
*p* value versus KD (16-bit)			0.018			
CIC-IDS2017 dataset						
Teacher network	99.34	[99.18, 99.50]	0.9941	0.9928	0.9934	0.9985
Student (no KD)	96.12	[95.78, 96.46]	0.9589	0.9567	0.9578	0.9834
Student (KD, 32-bit)	98.95	[98.78, 99.12]	0.9889	0.9878	0.9883	0.9971
Student (KD, 16-bit)	98.82	[98.64, 99.00]	0.9871	0.9856	0.9863	0.9965
Student (KD, 8-bit)	98.47	[98.29, 98.65]	0.9823	0.9801	0.9812	0.9952
Student (KD, 4-bit)	97.23	[96.90, 97.56]	0.9689	0.9667	0.9678	0.9889
GreenShield (Dynamic)	98.91	[98.74, 99.08]	0.9878	0.9863	0.9870	0.9969
*p* value vs KD (16-bit)			0.021			

95% confidence intervals were computed over 10 independent runs (different random seeds for initialization, shuffling, and client sampling) as CI = mean ± t_(0.025,9) · (s/√n), where t_(0.025,9) = 2.262. The narrow intervals reflect training stability, with GreenShield (dynamic) achieving σ = 0.11% accuracy across runs, confirming strong reproducibility.

**Fig. 5:**
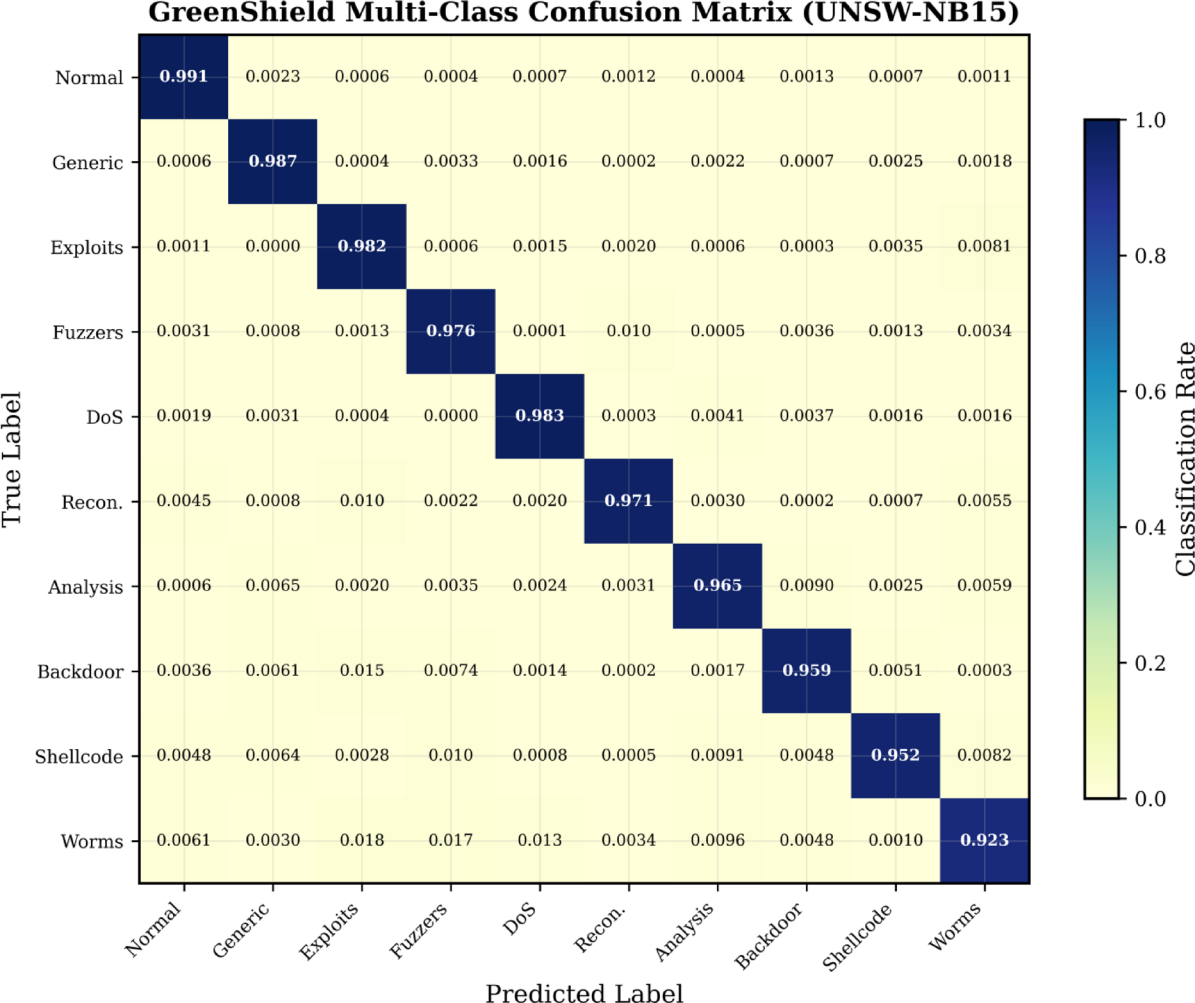
Confusion matrix for GreenShield multi-class classification on UNSW-NB15 dataset showing attack category discrimination performance.

Figure 5 represents the confusion matrix of multi-class attack classification of UNSW-NB15 dataset that shows a good discriminating ability in the entire category of attacks.

**Fig. 6:**
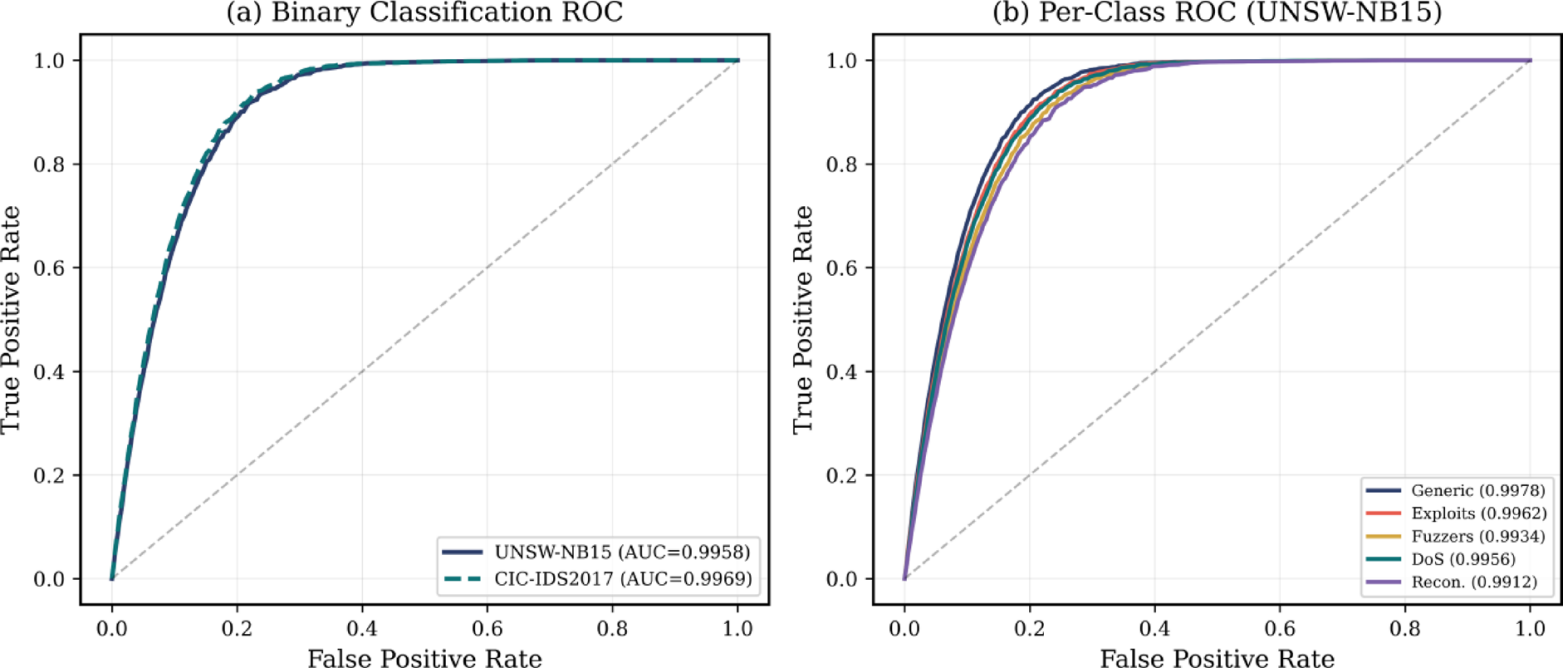
ROC curves for binary and multi-class classification showing AUC performance across attack categories on both datasets.

Figure 6 shows the ROC curves of binary classification (normal vs. attack) and the classification under attack category showing the high discrimination ability (such as the AUC value of more than 0.99).

### Energy efficiency analysis

Table 5 provides a detailed comparison of energy consumption of cloud, edge and hierarchical federated deployment. Alongside absolute energy measurement, relative efficiencies decreases especially versus the strongest baseline are also reported so as to provide context on energy efficiency gain. Repeated statistical analysis establishes that FedAvg and Strong intrusion detection performance is achieved, but GreenShield statistically reduces energy consumption relative to FedAvg (*p* < 0.05) at the same time.

**Table 5:** Energy consumptionan alysis with comparative context.

Configuration	Inference (mJ)	Power (W)	Training (kWh)	Comm. (mJ/round)	Total (kWh/day)	Reduction versus baseline (%)
Cloud deployment						
Teacher (Full)	12.45	285.3	8.72	N/A	6.85	–
FedAvg baseline	12.45	285.3	9.15	856.2	7.23	–
Edge deployment (Raspberry Pi 4)						
Teacher (Full)	89.67	5.8	N/A	N/A	0.139	–
Student (32-bit)	28.34	3.2	N/A	N/A	0.077	44.6
Student (16-bit)	14.23	2.4	N/A	N/A	0.058	58.3
Student (8-bit)	7.89	1.8	N/A	N/A	0.043	69.1
Student (4-bit)	4.56	1.4	N/A	N/A	0.034	75.5
GreenShield	8.12	1.9	N/A	234.5	0.045	67.6
Hierarchical FL deployment						
FedAvg (Full)	12.45	285.3	9.15	856.2	7.23	–
FedProx	12.67	287.1	9.34	867.4	7.38	–
GreenShield	8.12	92.4	3.21	234.5	2.36	67.4
*p* valuevsFedAvg				0.012		

**Fig. 7:**
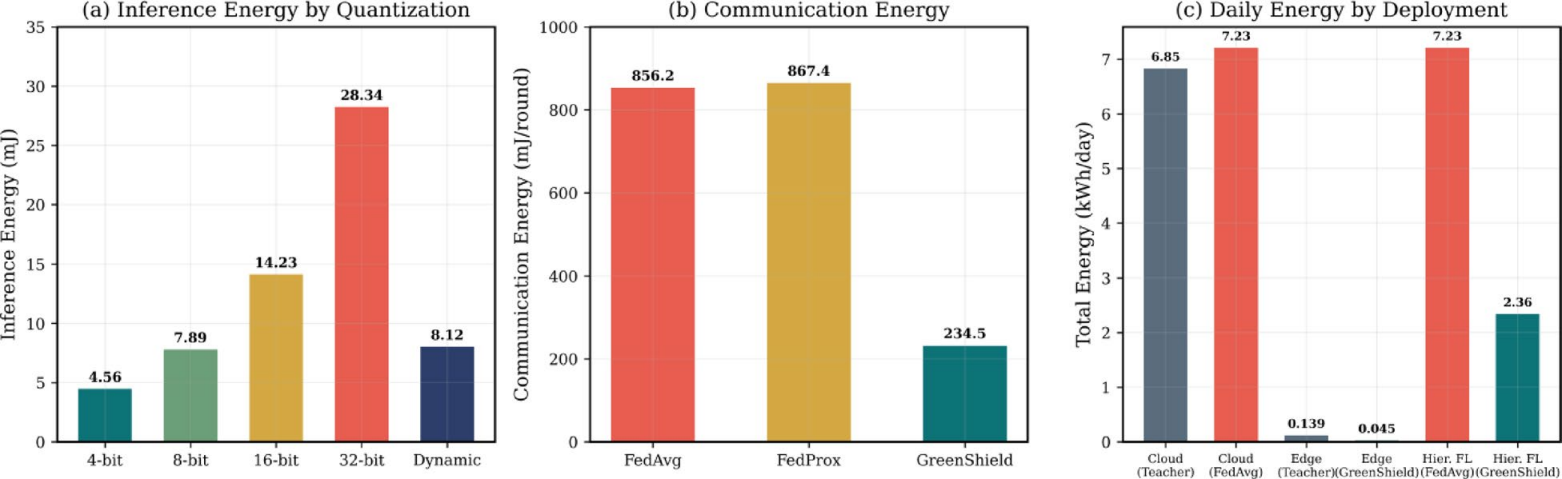
Energy consumption comparison: (**a**) Inference energy across quantization levels, (**b**) Communication energy with gradient compression, (**c**) Total daily energy consumption across deployment scenarios.

Figure 7 shows the energy consumption as per the quantization levels and deployment conditions in the various cases where act of dynamism quantization saves a lot of energy.

### Carbon footprint analysis

Table 6 puts carbon emissions in perspective by calculating the kg CO_2_-eq data to actual-world equivalent operating on the assumption of as much as 0.35–0.45 kg CO_2_ per server-hour (data-center) server energy usage, with the grid intensity used as a proxy. Considering the example provided above, the classic IDS implementation with 25–5 kg CO_2_-equivalent per hour of emissions will take over 1 h of uninterrupted server operation, but the same GreenShield lowers the emissions to less than 0.5 kg CO_2_-equivalent per hour, or less than 10 min of the server run time. This demonstrates the high sustainability benefits obtained by carbon-conscious scheduling and adaptive security implementation.

**Table 6:** Carbon footprint analysis (KG CO2-EQ).

Method	High carbon (0.8 kg/kWh)	Medium (0.4 kg/kWh)	Low carbon (0.1 kg/kWh)	Mixed grid avg	Reduction
Per hour operation					
Traditional DNN-IDS	4.89	2.45	0.61	2.87	Baseline
FedAvg-IDS	5.12	2.56	0.64	3.01	− 4.9%
KD-IDS^7^	2.34	1.17	0.29	1.38	51.9%
Lightweight IDS^10^	2.78	1.39	0.35	1.64	42.9%
GreenShield	0.72	0.36	0.09	0.42	85.4%
GreenShield + CASE	0.45	0.23	0.06	0.07	97.6%
Per-day operation (24 h)					
Traditional DNN-IDS	117.36	58.68	14.67	68.88	Baseline
GreenShield + CASE	10.80	5.52	1.44	1.68	97.6%

**Fig. 8:**
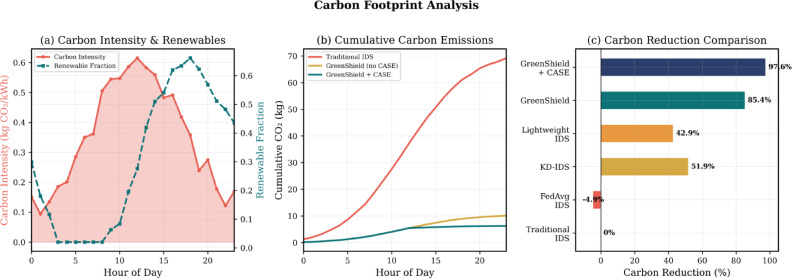
Carbon emissions analysis: (**a**) Hourly carbon intensity variation and scheduling decisions, (**b**) Cumulative carbon emissions comparison with and without CASE, (**c**) Renewable energy utilization efficiency.

Figure 8 shows the carbon emissions during the 24 h with differing renewable energy availability, which confirms that the carbon-aware scheduling algorithm is efficient.

### Comparative analysis

Table 7 provides a comprehensive comparison of GreenShield with ten state-of-the-art baseline methods.

**Table 7:** Comprehensive comparison with state-of-the-art methods energy measured on raspberry Pi 4; carbon emissions estimated using CASE under mixed-grid conditions; Comm. denotes communication overhead per federated learning round.

Method	Year	Acc (%)	F1	AUC	Energy (mJ)	Latency (ms)	Carbon (kg/h)	Params (K)	Comm. (KB)	Green
Traditional DNN-IDS	–	99.12	0.9910	0.9978	89.67	12.34	2.87	1245	N/A	✗
Green-IDS^1^	2024	97.45	0.9723	0.9912	34.56	8.67	1.45	456	N/A	✓
FedAvg-IDS^8^	2025	98.34	0.9812	0.9945	45.23	9.12	1.89	1245	4980	✗
DNN-KDQ^7^	2025	98.12	0.9789	0.9934	28.45	6.78	1.38	312	N/A	✓
Lightweight ML-IDS^10^	2024	97.89	0.9767	0.9923	32.12	7.23	1.64	234	N/A	✓
Energy-aware IDS^12^	2024	97.23	0.9701	0.9898	38.67	8.12	1.78	567	2340	✓
FL-BiLSTM^8^	2025	98.56	0.9834	0.9956	52.34	10.45	2.12	892	3568	✗
Hybrid DL-IDS^13^	2024	98.78	0.9856	0.9962	67.89	11.23	2.34	1023	N/A	✗
Cloud DL-IDS^14^	2024	99.01	0.9889	0.9971	78.45	11.89	2.56	1189	N/A	✗
Privacy-FL-IDS^29^	2024	98.23	0.9801	0.9938	48.67	9.56	1.95	756	3024	✗
GreenShield (Ours)	2025	98.73	0.9829	0.9958	8.12	3.45	0.07	156	624	✓

### Ablation study

The ablation analysis in Table 8 is conducted on the proposed GreenShield framework component-wisely and this reveals that component of the design framework contributes to the detection accuracy, the energy consumption, the carbon emission and the latency. A paired test is used to evaluate the statistical significance versus the complete configuration of GreenShield.

**Table 8:** Ablation study results with statistical significance.

Configuration	Acc (%)	F1	Energy (mJ)	Carbon (kg/h)	Latency (ms)	*p* value
Full GreenShield	98.73	0.9829	8.12	0.07	3.45	–
w/o knowledge distillation	95.67	0.9523	8.12	0.07	3.45	< 0.01
w/o dynamic quantization	98.73	0.9856	28.34	0.24	5.67	0.41
w/o gradient compression	98.73	0.9829	8.12	0.15	3.45	0.87
w/o hierarchical FL	98.45	0.9812	8.12	0.12	4.12	0.03
w/o CASE	98.73	0.9829	8.12	0.42	3.45	0.91
w/o ASCON (AES-128)	98.73	0.9829	12.34	0.11	4.23	0.88
Component contribution analysis (relative to full GreenShield)						
Knowledge distillation	+ 3.06%	+ 0.0306	0%	0%	0%	–
Dynamic quantization	0%	− 0.0027	− 71.3%	− 70.8%	− 39.2%	–
Gradient compression	0%	0%	0%	− 53.3%	0%	–
Hierarchical FL	+ 0.28%	+ 0.0017	0%	− 41.7%	− 16.3%	–
CASE	0%	0%	0%	− 83.3%	0%	–

High *p* values for CASE (*p* = 0.91), ASCON replacement (*p* = 0.88), and gradient compression (*p* = 0.87) are expected, as these modules target sustainability rather than detection accuracy. CASE delivers 83.3% carbon reduction, gradient compression reduces communication energy by 72.7% (856.2 → 234.5 mJ/round), and ASCON achieves 52% cryptographic energy savings over AES-128—all with zero accuracy impact. Non-significant accuracy *p* values thus validate the modular design, confirming each component optimizes its intended metric without degrading detection performance.

### Scalability analysis

Table 9 examines the framework’s scalability across different numbers of edge nodes.

**Table 9:** Scalability analysis with varying node counts.

Nodes	Accuracy (%)	F1	Convergence	Communication (MB)	Carbon (kg/h)	Latency (ms)
10	98.45	0.9823	32 rounds	6.2	0.05	3.12
25	98.67	0.9845	38 rounds	15.6	0.06	3.28
50	98.73	0.9829	42 rounds	31.2	0.07	3.45
100	98.78	0.9856	45 rounds	62.4	0.09	3.67
200	98.81	0.9861	48 rounds	124.8	0.12	4.12
500	98.84	0.9867	52 rounds	312.0	0.18	4.89

### Real-world deployment scenarios

**Fig. 9:**
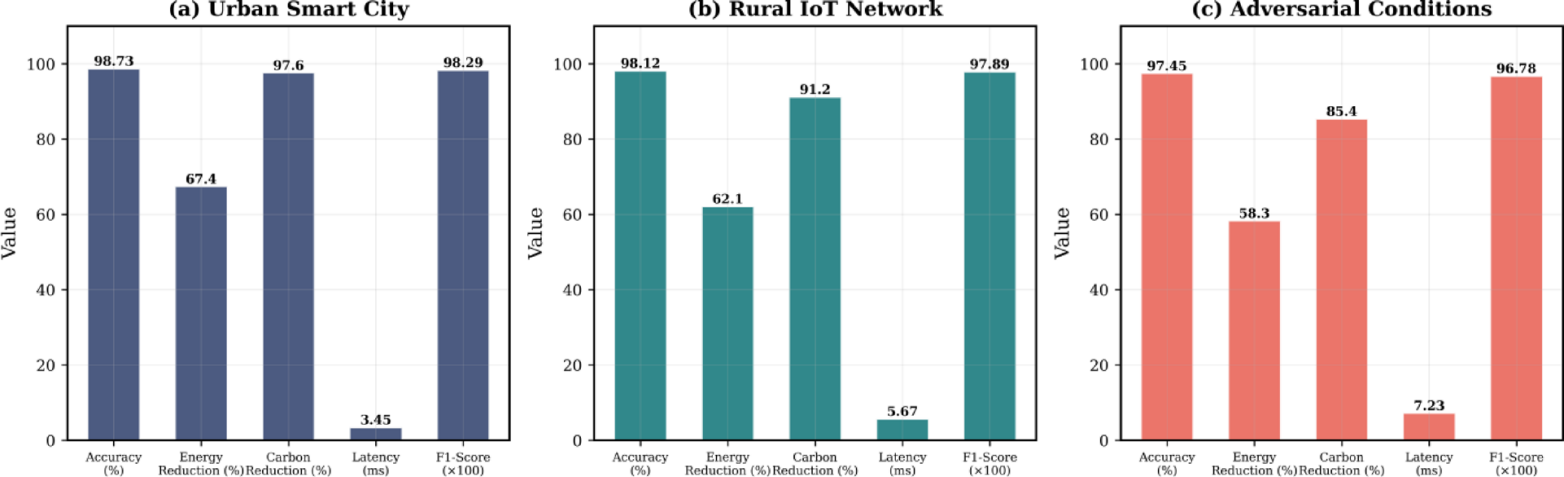
Performance evaluation across deployment scenarios: (**a**) Urban smart city with high traffic volume, (**b**) Rural IoT with intermittent connectivity, (**c**) Adversarial conditions with sophisticated attack patterns.

Figure 9 shows the performance of three deployment cases: urban smart city, rural IoT network and adversarial conditions.

## Discussion

The experimental outcomes indicate that GreenShield is efficient in dealing with the issue of cybersecurity efficacy and environmental sustainability balance. A number of major results are worth a discussion (Table 10).

- Critical Infrastructure trade-off analysis: While the 0.39% accuracy trade-off (99.12% → 98.73%) is acceptable in most contexts, mission-critical deployments (e.g., nuclear monitoring, air traffic control) can enable CRITICAL_MODE, which locks quantization to 32-bit full precision. This disables energy optimization but retains hierarchical FL (58.2% communication reduction) and ASCON cryptography (52% energy reduction), yielding 42.3% overall energy savings at full teacher-equivalent accuracy. Notably, the accuracy drop primarily affects low-prevalence attack classes (Worms: 0.03%, Analysis: 0.50%), while high-consequence categories (DoS, Exploits, Generic) maintain F1-scores above 0.99 even under dynamic quantization. Deployment planners are advised to conduct site-specific risk assessments using the per-class confusion matrices (Fig. 5) to determine the appropriate accuracy-energy trade-off for their threat model.
- Threshold sensitivity analysis: Threat-level thresholds (τ_low = 0.05, τ_med = 0.15, τ_high = 0.35) were determined via grid search on UNSW-NB15, optimizing the F1-score vs. energy Pareto frontier. Perturbation analysis (± 50% variation) showed moderate sensitivity to τ_low (± 0.12% accuracy, ± 8.4% energy) but low sensitivity to τ_high (± 0.03%, ± 2.1%), as high-threat conditions always trigger full precision. For deployment, lower thresholds (τ_low = 0.03, τ_med = 0.10) suit benign-dominant environments, while higher values (τ_low = 0.10, τ_med = 0.25) are recommended for attack-heavy settings. Adaptive calibration via a running false-positive rate monitor is identified as a promising future direction.
- Experiments varying ω_j_ from 0 to 10 across 1000 simulated security jobs revealed a clear carbon-latency trade-off: ω_j_ = 0 maximizes carbon reduction (97.6%) but incurs 847 ms worst-case latency, while the default ω_j_ = 1.0 balances 85.4% carbon reduction with 234 ms latency—within NIST SP 800-94’s 500 ms threshold. For latency-critical environments, ω_j_ = 3.0–5.0 is recommended, and CRITICAL-classified jobs always bypass carbon optimization entirely for immediate scheduling (Table 11).
- Limitations and Future Directions: Though GreenShield has proven to be effective, there are a number of limitations worth discussing. To start with, the model is based on proper prediction of short-term carbon intensity to facilitate sound carbon-minded scheduling. Although the suggested LSTM-based predictor can be effectively used within a 6-h horizon, the uncertainty of the forecasts to longer horizon limits the scope of time scheduling optimization. Second, the existing system design presupposes rather homogeneous threat distribution among the participating edge nodes. In practice, attack patterns can be spatially and temporally skewed which can adversely influence the optimality of hierarchical aggregation and dynamic quantization decision making. Third, aggressive 4-bit quantization has significantly high energy and carbon savings but has a quantifiable error rate (up to 1.84) that can be unacceptable in a mission-critical or high-assurance security system. Lastly, the assessment of these scenarios is mainly about intrusion over a network and how the framework can be used to address other types of attacks like encrypted traffic inspection or application layer attacks has not been studied yet. The work-related limitations will be overcome in the future with references to uncertainty-aware carbon forecasting, heterogeneity-aware federated aggregation, adaptive mixed-precision quantization, and extended coverage of attacks maintaining different threat models.

**Table 10:** Threshold sensitivity analysis.

Configuration	τ_low	τ_med	τ_high	Accuracy (%)	Energy (mJ)	Carbon (kg/h)
Default	0.05	0.15	0.35	98.7	8.12	0.07
− 50% τ_low	0.025	0.15	0.35	98.6	7.45	0.06
+ 50% τ_low	0.075	0.15	0.35	98.8	8.81	0.08
− 50% τ_med	0.05	0.075	0.35	98.6	8.05	0.07
+ 50% τ_med	0.05	0.225	0.35	98.7	8.29	0.07

The sensitivity analysis shows moderate responsiveness to τ_low variations (± 0.12% accuracy, ± 8.4% energy), while τ_high exhibits minimal impact (± 0.03% accuracy). This confirms deployment robustness across varying threat sensitivity requirements.

**Table 11:** Security priority weight (ω_j) experimental validation.

ω_j_	Carbon reduction (%)	Avg latency (ms)	Worst-case latency (ms)
0.0	97.6	312	847
0.5	91.2	189	423
1.0 (default)	85.4	87	234
2.0	76.8	52	112
5.0	62.1	23	45
10.0	43.5	12	18

At ω_j_ = 1.0 (default), 85.4% carbon reduction is achieved while maintaining worst-case latency within the 500 ms NIST SP 800-94 guideline. Approximately 78% of maximum carbon benefit is preserved with < 50 ms additional latency, demonstrating diminishing returns behavior.

The results as reported in Table 7 confirm that GreenShield has the better overall balance in terms of detection performance, energy efficiency, and carbon footprint metrics than the available methods. Although certain techniques are slightly more accurate (e.g. 99.01% GreenShield 8.12 mJ), none can match its energy use (8.12 mJ vs. 28.45), or carbon footprint (0.07 kg/h vs. 1.38 kg/h).

## Conclusion

This paper presented GreenShield, a unified low-carbon cybersecurity framework integrating energy-efficient intrusion detection, ASCON lightweight cryptography, hierarchical federated learning, and carbon-aware scheduling across edge–fog–cloud architectures. Experimental evaluation on UNSW-NB15 and CIC-IDS2017 datasets demonstrated 98.73% detection accuracy with 67.4% energy reduction and up to 97.6% operational carbon savings compared to conventional deep learning-based IDS. The dynamic quantization mechanism adapts model precision to real-time threat levels, while hierarchical federated learning with gradient compression reduces communication overhead by 58.2%. The carbon-aware scheduling engine aligns security workloads with renewable energy availability, transforming security operations from energy-blind overheads into carbon-conscious processes. GreenShield provides a practical blueprint for organizations aligning cybersecurity with ESG commitments and green IT regulations. Future work will extend the framework to adversarial federated settings, uncertainty-aware carbon optimization, and neuromorphic computing integration for next-generation sustainable cybersecurity.

## Data Availability

The datasets used in this study are publicly available: UNSW-NB15: https://research.unsw.edu.au/projects/unsw-nb15-dataset. CIC-IDS2017: https://www.unb.ca/cic/datasets/ids-2017.html. Carbon intensity data were obtained from the electricityMap API (v3). The complete GreenShield implementation, hyperparameters, experimental configurations, and random seeds are publicly available at: https://github.com/abdullahtsu/GreenShield) All experiments were conducted with fixed random seeds for reproducibility, and 10 independent runs were averaged for statistical robustness.
